# It’s all about the patients: a shift in medical students’ approach to learning during a novel distributed integrated clinical rotation

**DOI:** 10.1186/s12909-024-06112-8

**Published:** 2024-10-15

**Authors:** Ian Couper, Susan van Schalkwyk, Julia Blitz, Therese Fish, Kobus Viljoen, Luné Smith

**Affiliations:** 1 Division of Rural Health (Ukwanda), Department of Global Health, Faculty of Medicine and Health Sciences, Stellenbosch University, Cape Town, South Africa; 2 Department of Health Professions Education, Faculty of Medicine and Health Sciences, Stellenbosch University, Cape Town, South Africa; 3 Clinical Services and Social Impact, Faculty of Medicine and Health Sciences, Stellenbosch University, Cape Town, South Africa; 4 Division of Family Medicine and Primary Care, Department of Family and Emergency Medicine, Faculty of Medicine and Health Sciences, Stellenbosch University, Cape Town, South Africa; 5 MBChB Programme, Faculty of Medicine and Health Sciences, Stellenbosch University, Cape Town, South Africa

**Keywords:** Distributed training, Integration, Learning experience, Clinical training, Patient-based, Context, Relationships, Participation, Transformation

## Abstract

**Introduction:**

To ensure that pre-final year medical students at Stellenbosch University were able to resume clinical training during the COVID-19 pandemic, a 12-week integrated rotation was introduced, during which students were distributed across a widespread training platform in two provinces of South Africa, utilizing a range of health care facilities in both rural and urban areas, rather than the central academic hospital (CAH) in which they would have been doing clerkships. Called the Integrated Distributed Engagement to Advance Learning (IDEAL) rotation, this clerkship was based on supervised engagement in healthcare services, focusing on patient-based clinical training, self-regulated learning and student participation as integral members of clinical teams. The success of this emergency intervention has led to its formal incorporation into the medical curriculum. This study aimed to understand the factors that influenced learning among students undertaking the IDEAL rotation at multiple sites on a distributed training platform.

**Methods:**

Using an interpretive paradigm, we sought to conduct focus group interviews with students who completed the first iteration of the IDEAL rotation in the year after they had undertaken it to understand their experiences. All 252 students who were eligible were invited to participate by email on several occasions. Ultimately three focus group discussions and two individual interviews were undertaken, based on volunteers. Using a semi-structured interview guide, these explored student perceptions of their learning and growth through the rotation. Inductive and deductive analysis was carried out to identify themes.

**Findings:**

Student descriptions of their learning experiences coalesced in 6 themes. The rotation was an *enabling learning experience*, which was more practically focused and assisted students in developing confidence in their clinical skills. It was seen to be a *humanizing learning experience* with greater opportunities for the development of relationships with patients and families, as well as with health professionals, who made them feel part of the team, so it was also a more *collegial learning experience*. At the same time, it was a *variable learning experience* with a lack of standardization on a number of levels and challenges being experienced at particular sites regarding both logistics and the nature of the exposure. Students perceived it to be a very *different learning experience* from what they had encountered in the CAH in terms of relationships, the kinds of patients and problems they saw, and their active participation. Through this, they also learned more about themselves and their roles, making it a *personal learning journey*. The findings confirm the importance of the dimensions of person, participation and place for being and becoming a doctor in a clinical environment.

**Conclusions:**

Student learning experiences in the IDEAL rotation emphasize the importance of context, reinforcing the value of a distributed training platform in developing health professionals who are responsive to their environment. They emphasize the vital role of active participation in learning and the centrality of relationships in medical training, helping to develop graduates who are human beings and not only human doings.

**Supplementary Information:**

The online version contains supplementary material available at 10.1186/s12909-024-06112-8.

## Background

It has become axiomatic to argue that it ‘took a pandemic’ to shift many entrenched teaching practices, including those in health professions education. The impact of COVID-19 on the teaching and training of medical students has been extensively documented with publications on the topic emanating from all over the globe. Some early examples emanated from Australia [1]; Greece [2]; Jordan [3]; Turkey [4]; the UK [5]; and the USA [6]. The situation has been described as challenging, characterised by innovation and opportunity by some [2, 7], but also in the context of clinical training described as a ‘recipe for failure’ [5]. This article seeks to contribute theoretically to the global conversations regarding the impact COVID-19 had on teaching and training by showcasing and analysing the innovation required to allow clinical training of senior medical students to continue despite the challenges the pandemic posed. This was achieved by implementing a novel, longitudinal, integrated rotation across a distributed platform.

The medical (MBChB) programme of the Stellenbosch University (SU) Faculty of Medicine and Health Sciences (FMHS) is a six-year undergraduate entry course that prepares students for internship and registration as medical practitioners within the South African context. The clinical years are structured into three phases. The last phase, student internship, spans 16 months. It starts with the second semester of the fifth or pre-final year (August) and ends at the conclusion of the second semester of the sixth or final year (November). Most of the clinical rotations are undertaken at a tertiary public health facility, the Central Academic Hospital (CAH), which is the institution’s major teaching site, except the four week primary health care rotation undertaken across a wide range of sites on the distributed training platform.

In 2020, at the start of the COVID-19 pandemic, clinical services at the CAH were reorganised, with many of its medical staff being redeployed. To assist this and to comply with national disaster regulations [8], the FMHS temporarily withdrew all students from the clinical platform. Once the adapted clinical situation felt more stable, negotiations between the FMHS and the Department of Health led to final year students being allowed back into the CAH (Dr Therese Fish, personal communication). This left a challenge as to how to enable the pre-final year students to commence their student internship clinical training so that their graduation in 2021 would not be delayed. In response to this challenge, the Integrated Distributed Engagement to Advance Learning (IDEAL) rotation was designed [9]. IDEAL was a 12-week, integrated, clinical-focussed rotation for students, based on our prior experiences in the Rural Clinical School [10], in developing a framework for distributed training as part of a SU-led national project [11], and in implementing an integrated primary care rotation at the University of the Witwatersrand, also in South Africa [12, 13].

The design team consisted of colleagues from the FMHS management, academic staff, the Rural Clinical School, the logistics team as well as student representatives. Students needed to be placed outside of the CAH and distributed across the breadth of the clinical training platform^1^ in the Western and Northern Cape provinces of South Africa. Sites identified were district and regional hospitals with their primary care clinics in urban, peri-urban, and rural settings. The 4-week Primary Health Care rotation in the student internship formed the building block for IDEAL. Other specialty rotations (General Surgery, Internal Medicine, Paediatrics, Obstetrics and Gynaecology, Psychiatry and Orthopaedic surgery) were realigned by moving learning outcomes which could be addressed through opportunities on the distributed platform, and the time allocated to achieving them, into IDEAL; this shortened each by 1–2 weeks, enabling us to create the 12 week IDEAL rotation.

An important design component was that the students would join teams and contribute to service delivery at the sites, which included supporting COVID-19 responses, for which they received specific infection, prevention and control (IPC) and personal protective equipment (PPE) training. Clinical staff were asked only to provide supervision and “bedside” training of students, so as not to hinder their work. No formal tutorials or assessments were to be performed by the clinical staff. This required a significant shift by students to taking supervised responsibility for patient care and becoming integral members of clinical teams. The rotation was structured as alternating weekdays, one on the clinical platform and the next which students could use to complete assigned learning tasks, such as logging patient encounters, writing reflective learning pieces, and working on group assignments (quality improvement project and evidence-based medicine case study). In the planning we recognised that these shifts would probably require the students to develop more self-regulated and self-directed learning habits.

Resources (slideshows and pdf notes) that previously had been made available at lectures and tutorials were placed online in the students’ Learning Management System (SUNLearn). The students logged patient encounters on a mobile app (VulaMobile) using a modified SNAPPS [14] template. Medical practitioners appointed as online learning facilitators then assisted students to explore clinical reasoning through these patient encounters. Wellness supporters (from various disciplines and professions) were also appointed to check-in with students, basing their conversations on reflective pieces submitted online by their group of students. These supporters served as a sounding board for the students as they encountered new clinical experiences and journeyed into new ways of learning. This was particularly important because students received very little specific preparation for the rotation. Students returned to the CAH after the IDEAL rotation to complete the final speciality rotations of their student internship. These rotations continued as they had (albeit slightly shortened) prior to this pandemic-induced innovation.

Developing the IDEAL rotation de novo provided a unique opportunity to research the innovation as it was being developed. The constraints of timing meant that the process of development and implementation were running concurrently and iteratively. The module team needed to learn from these processes and make adaptations along the way as an absolutely necessary ingredient of implementing the module. Furthermore, the possibility of the module becoming a key feature of the MBChB programme going forwards, as has subsequently occurred, was raised at the outset. This made it even more crucial to learn lessons from the initial real-world experiment while reflecting on these lessons in light of existing research.

## Theoretical framework

**Fig. 1:**
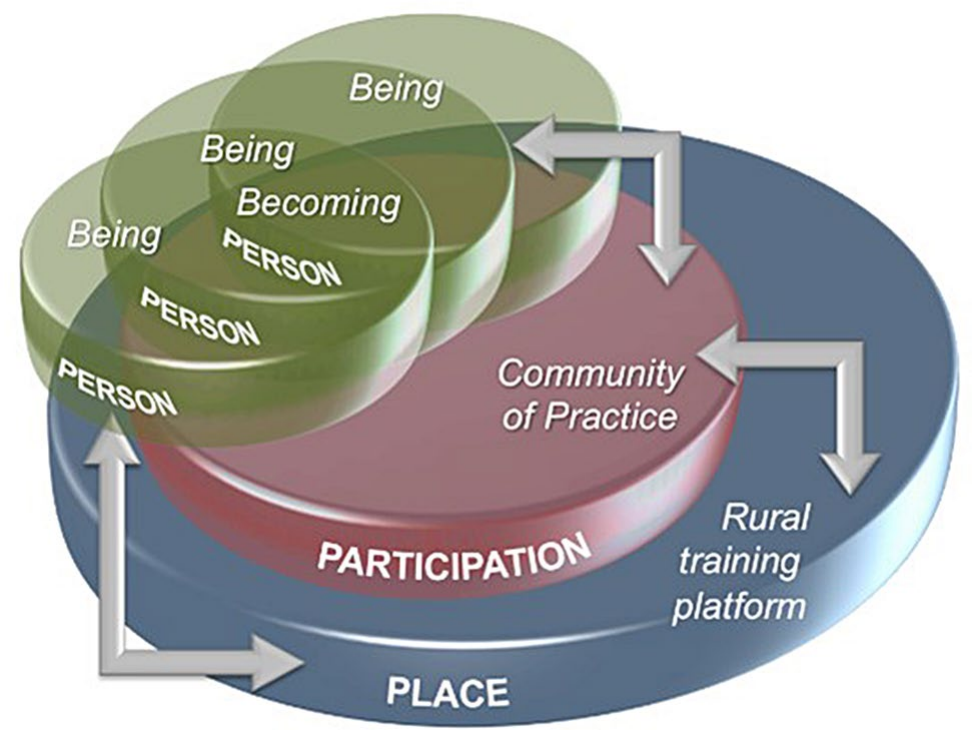
Being and becoming a doctor, taken from Van Schalkwyk et al. 2015 [17]

Clinical training that occurs at distributed sites away from the traditional tertiary hospital has been acknowledged as offering unique transformative learning experiences [11, 15]. This work emphasizes the importance of these sites [15] and their concomitant benefits in terms of the affordances they offer [16]. Our own work in this area proposed a model for being and becoming a doctor in which we centred the student (person) within a particular site (place), where they could legitimately engage within the community of clinical practice (participation) (Fig. 1).

In this article, we report our analysis of the students’ perspectives on their learning experiences and growth garnered while evaluating this innovation in light of this theoretical model. Our specific research question was: what factors influenced learning among students undertaking the IDEAL rotation at multiple sites on a distributed training platform? Our intention with this work is to reflect on the lessons that the experience- and evidence-driven rapid design of a rotation (clerkship) necessitated by COVID-19 offers us in responding to longer-term demands to broaden health professions training by using an extensive distributed training platform.

## Methodology

The study was situated within an interpretive paradigm as we sought to derive knowledge of the IDEAL rotation from the students’ described learning experiences [18], using an exploratory qualitative design. This was part of a larger mixed methods study using the over-arching principles of Educational Design Research [19].

To generate our data, focus group interviews were conducted with students during 2021 while they undertook their final disciplinary rotations at the CAH. All 252 students in the final year class who participated in IDEAL in 2020 were invited by email to participate in interviews. Five focus groups involving four to six students were planned. Three focus groups involving 10 students and a further two individual interviews were conducted based on student responses. The individual interviews were conducted because additional focus groups were arranged but only one student arrived for each. The focus groups and individual interviews were carried out by an experienced researcher (IC) with the support of a research assistant. A semi-structured interview guide developed by the research team (see supplementary file) explored student perceptions of their learning during IDEAL. Focus group discussions and interviews were audio-recorded with the informed consent of the participants, and transcribed verbatim by the assistant. Focus group participants were requested to respect and maintain confidentiality.

Two members of the research team (SvS and IC) carried out the analysis. An initial ‘soft focus’ analysis [20] of the transcripts enabled familiarisation with the data to get a sense of the students’ experiences overall. Inductive coding was done individually and then compared to identify themes. Thereafter, we deductively applied the model for being and becoming a doctor to the identified themes.

All participating students gave informed consent, and data (transcripts) were anonymised. The Social, Behavioural, and Education Research Ethics Committee (Project no. 18587) approved the research. SU institutional permission (ref. IRPSD-1889) and permission of the MBChB Programme Committee to conduct the research were also obtained.

## Findings

Twelve sixth-year students who had completed the IDEAL rotation during 2020 were interviewed in three focus groups and two individual interviews. These students had completed IDEAL at rural and urban sites in both the Western Cape and Northern Cape provinces, including regional hospitals, as well as large and small district hospitals. Learning occurred in many ways, and there was some variability in the way students described their experiences, but the responses were mostly positive and appreciative. The findings are initially presented according to six themes obtained from inductive analysis that illuminate different aspects of their learning experience.

### An enabling learning experience

For almost all respondents, the IDEAL rotation manifested as an enabling learning experience, one that strengthened their learning. Students described the sites as ‘*great for learning*.’ Teaching was described as ‘*hands-on*’. Almost without exception, students described being more confident about their clinical skills after the IDEAL rotation.

*The thing that I always think about IDEAL is confidence. I feel like being there really built my confidence and my friends who I’ve been with too. So when we started there and we ended we were completely different people in the way that we presented*, *the way that we came up with plans*, *in the way we’ve managed patients. (FG1)*

The enabling experience described by students was not only the learning opportunities offered at the IDEAL sites, and the growth in their knowledge and skills that resulted from these, but also a different way of knowing, moving from ‘do I know the answer to this question?’ to ‘what am I going to do?’ Several spoke about learning independently with an ‘incredible amount of autonomy’, developing a management plan, and emphasizing the value of longitudinal exposure.

*I think in [regional hospital] there are one or two departments that are very geared towards students*, *so I think a bit more likely say where the doctor would refer a patient and be like*, *oh this is a great case go and see that patient*, *but in other [departments] it was very much self-directed and I quite appreciate the variety of that*, *where you were able to gain an approach in a self-directed way to sort of streamline your own learning and take away … So learning to sort of gauge what is the most important to take away from a specific case*, *that was something brand new that I think this prolonged exposure on the IDEAL rotation really*, *really helped. (FG2)*

It was also clear that students were often expected to work independently, particularly at some of the more rural sites. This emphasized how it taught them to be less reliant on their supervisors.

*At [rural hospital] we could work very independent so the first few weeks we sort of still have the [CAH] mentality where we go there and we are like ‘ok doctor can I see this patient*, *is it fine if I draw bloods*, *is it fine if I…’*, *whereas towards the end of the rotation it got busy at EC and the doctor on call would get busy and one of us would be on the other side basically running the EC with the doctor. (FG5)*

Most appeared to embrace these self-directed learning opportunities, following up on interesting patients they had seen, but some felt that they had been left to teach themselves. Here the role of the supervisor on site appeared to be important. One student described how they enjoyed not having ‘*someone watching over you’*, but with the caveat that they had felt secure in the knowledge that ‘*someone’s got your back*’.

Some students found it difficult to adjust to the absence of learning opportunities that had been structured by others. While some explained that at some sites they were allowed to ‘*go to work*’ as they wished, this came with the cautionary that they needed to set clear boundaries to ensure that they balanced service delivery with their own learning. They described feeling concerned about ‘*missing out on academics*’, which most seemed to associate with tutorials during rotations at the CAH. Others suggested they would have valued more opportunities to consolidate their learning and asked for more structure and better preparation for the rotation.

*I’m somebody who’s fine with taking initiative and teaching myself and I can do that but it’s exhausting trying to do the work; like do the studying*, *doing the work and trying to teach yourself as well like it’s very frustrating especially when you don’t have anybody you can speak to like. (FG4)*

The students’ comments made it clear that the IDEAL rotation was ‘*not easy*’. While some experienced it as less pressurized, others described being exhausted, criticizing the number of assignments, the absence of structured tutorials, and the lack of feedback.

*The pressure of the assignments and the things kind of took away some of the fun experience of IDEAL. (FG1)*

Nevertheless, overall the feeling was that the rotation was pivotal in preparing them for internship.

*I think that the time*, *it was mostly just practical knowledge*, *so it was a lot of hands on practical foundational knowledge. [CAH] rotations kind of follows you up academically*, *but the IDEAL rotation really prepares you kind of practically almost like street-smart medicine. Like*, *what you need to do*, *what you need to know*, *what you need*, *don’t miss. What are the red flags? I feel like it’s definitely good to prep you for internship. I feel like otherwise you could feel that gap like you have all this academic knowledge but not necessarily the skills*, *or just the general knowledge to try and approach a patient like from the basics. (FG2)*

*It was the best 12 weeks I’ve had in the last 6 years of medical school in terms of what you learn*, *independency*, *and knowing that if a child comes in crying it’s oh so he probably has a middle ear infection not 30 other things that you think and you don’t know what. (FG5)*

### A humanising learning experience

A dominant perspective among the respondents related to how the IDEAL experience felt like a more ‘*humane experience*’ allowing them to ‘*be human*’. This played out in different ways but generally appeared to be catalyzed by the opportunity to follow up on patients and being given (and taking) responsibility for ‘their’ patients. Having more time to spend with their patients enabled the development of relationships with not only the patient but often also the families of these patients. The patient influence was a golden thread running through the study and strongly linked to the ‘humanising’ experience. In this instance, getting to know the patients as a result of the longer rotation period created the opportunity to build relationships, but also to see a particular (learning) cycle being completed.

*I think it’s also nice when your patients come back … if a patient came in week 1*, *I saw them till week 12*, *like I knew he was gonna come back for the cast to be removed or needed new dressings*, *who we should follow up or who we need to wait for them to come back from ultrasound in [referral hospital]. It was almost like you finally finished something that you start and the patient is not just a learning opportunity. (FG4)*

*… the goal of the rotation is not to learn all the work or study all the work. Now it’s more about*, *you see your patient and you learn from your patient. (PG5)*

*And I think I had so many patients that taught me so much too and had so many opportunities to advocate for them*, *that would not necessarily have happened here so I*, *I got to speak to doctors and other hospitals*, *I got to speak to like toxicologists*, *I had to speak to*, *you know*, *Department of Health officials to organize transport for patients*, *you know… (FG2)*.

Students described the patients as having become important to them and how this led to a mutually beneficial relationship.

*I think I like my patients; good relationships are big for me. So I think the fact that I now know how important that is for me. I think it’s important for them as well. You can see the patients*, *you recognize when you spend time with them*, *appreciate it even more. So they respected you more. (FG4)*

As mentioned above, the role of the supervising doctor at the site was a key influence.

*I remember when we went to the [primary care] clinic*, *very awesome*, *the doctor actually sat us down with a cup of coffee*, *the head doctor. I don’t remember who it was*, *and we just talked about where you wanna go next year*, *what you wanna do … I really got that from that doctor who oriented us even working at the clinic*, *um*, *it just feels like a humane medical experience. (FG3)*

*It was so great like we saw patients. The doctor asked them stuff about their family man*, *you don’t do that here [at the CAH]. No one cares*, *they just want like assessment, plan*, *you know. It was just nice*, *people*, *they are humans you know … This is so and so and these are her children and these are the names of her children. (FG3)*

Being recognized and ‘known’ themselves as individuals, not simply as ‘another student’, was also an important part of this.

*Whereas most of the time [in other rotations] mental health is not looked after; no one really cares about you as a person*, *you’re just a medical student…. [IDEAL] just feels like a humane medical experience. (FG3)*

### A collegial learning experience

Linked to the above, and often contributing to it, was the nature of the relationships between the students and the health professionals at the different sites. Most respondents described how, for the first time in their academic careers, they felt part of ‘*the team*’ in the hospital and/or clinic context. Students spoke about how they often felt their engagements with other healthcare professionals at the different sites were more collegial.

*There was a lot of times we actually just felt like actually part of the team managing the patient with the hospital. It was sort of like you’re in the hospital together. (FG1)*

*When we presented a patient*, *it was really like a discussion amongst colleagues (FG1)*

They enjoyed getting to know the other hospital staff and the sense that ‘*everyone knows you*’ in return. This was facilitated by the continuity resulting from being placed at the same site for three months.

*[Urban Hospital] was the best place ever. Um*, *the teaching was phenomenal*, *you learnt something every day*, *it was hands on so I did so many procedures*, *I did so many casts*, *and you really felt like part of the team. I think that was the biggest thing; they treated you like a colleague*, *and not like a student*, *you know*, *who’s kind of like asking for things and kind of bothering them*, *it didn’t feel like that at all. (FG3)*

Students also specifically commented on how they became more aware of the value of the multidisciplinary team, with several students describing instances where, for example, nurses had been instrumental in their learning.

*Just working in a way that allows other people to also work*, *so team work. You don’t have to like everybody but you do have to be able to work with them. That was a big thing*, *the value of good nursing staff. (FG4)*

*So*, *I think those clinical skills*, *especially with the Allieds. I think it was useful. Because I mean*, *like*, *I get to know the physio*, *I know who the dietitian is*, *I know the OT*, *so like I can’t really write a bad referral because they’re gonna know it’s coming from me. The gonna be like*, *are you serious? Did you really just refer a patient for pain? No*, *so there’s that in terms of clinical skill*, *and I think like the MDT involvement … communication*, *learning from them. Honestly there’s nobody you can refer to better than OT. (FG4)*

### A variable learning experience

The experience was not standard across all of the sites. A recurring theme across the interviews was that, while the students were generally positive and appreciative about the experience, there were challenges. The variability featured in the sites themselves (particularly with regards to logistics, the availability of Wi-Fi, the quality of the accommodation, the transport arrangements, and how the rotation had been structured) and the nature of the exposure (not all sites offering the same range of disciplines; not all having the same level of collegiality). The nature of the supervision was mentioned as being particularly variable across sites.

*Anyways there was a very big discrepancy between what various facilitators wanted from their students. (FG5)*

*At [regional hospital] I’m not sure if it was just the timing because it was the first IDEAL rotation*, *it felt like in one or two of the two disciplines that we rotated*, *it felt as if the doctors weren’t prepared to receive students so it did feel like we were sort of in the way. And maybe not as much interaction with the patients as we would have liked and yes like I said it might have just been growing pains getting that it was the first rotation that was just one thing which would be a bit disappointing. But with some of the departments it was a complete opposite*, *we just got sucked in and became part of the team from the get-go*, *that was just incredible. (FG2)*

*The variability in supervision is very common I think in the format that we use on IDEAL. Especially since the learning facilitator was not on site … The issue was that at our hospital there is one doctor who is involved with students but he went on leave for a month*, *so then we were left on our own … some doctors are not there to teach the students. Some don’t appreciate being asked questions by students. (FG2)*

Interestingly, however, while some students felt that the ‘rural’ sites had been more enjoyable than those in the urban and peri-urban sites, this position was not held by all, with some of these latter sites also being described as ‘*great*’.

### A different learning experience

Multiple differences between the learning experience at the IDEAL sites in comparison with previous rotations that they had already completed in the preceding two years (70–90 weeks) at the CAH were described. As seen above, the sense of continuity, both with patients and with hospital colleagues, was a key issue raised. They specifically referred to the ‘*patient-oriented*’ culture that they experienced during IDEAL as opposed to the CAH where they felt there was less opportunity to engage with patients over a period of time. Learning was much more likely to be patient-based (rather than side-room tutorials), which they enjoyed.

*So*, *this learning was more patient orientated … So I just felt like more what actually helping people is because they’re more than just diseases*, *or patient with the pre-eclampsia…. So it was nice*, *it felt*, *I guess it just feels human. (FG3)*

*So I guess that’s how I changed. I just tried to be more patient orientated in a more holistic way … it’s changed a lot how I talk to patients who are sick or scared or parents who were worried. (FG1)*

The nature and content of learning were also considered to be very different, with students reporting that patients presented with conditions that they felt they ‘*should be seeing*’, which represented ‘*the basics*’ of what they need to know as graduates. They highlighted the importance of being exposed to undifferentiated patients in terms of challenging their clinical reasoning instead of what happened at the CAH where ‘*you know what to look for*’.

*But then in [a rural hospital] we got patients who came in with a cough*, *they fell*, *or their tooth is sore*, *or like*, *what we’d consider simple complaints. And we wouldn’t necessarily see [these] at Tygerberg. So for me what I mainly focused on was going back to those complaints and learning how do I approach something like a toothache. Because the patient said their tooth is sore so I was like oh my god*, *what do I do*, *I was like*, *just give me a second*, *I’m gonna come back now. I literally took out my phone and I went to the PACK [guidelines]. And I looked for tooth pain because I was like*, *I don’t know what to do and came back. From that I realized afterwards*, *I could get this thing*, *it could be a dentist thing*, *it could be mouth thing*, *it could be an ear thing. Like*, *you don’t even realize it. It’s just a tooth but it’s all those structures that can cause that pain. So*, *for me it wasn’t about all the big things*, *it was actually going down to those complaints that was what the patient came into the EC with. (FG1)*

*The undifferentiated patient is a unique challenge outside of [CAH] because again in [CAH] everyone comes with a referral letter and many people have seen them already. (FG1)*

*It was still a very nice experience to be able to see patients early on in disease processes. (FG2)*

Students, particularly at the smaller sites, also commented on how they were given many more opportunities to practice certain procedures and be exposed to a wide range of patients. This was contrasted with the hierarchy at the CAH where, as medical students who were not yet student interns, they were far more removed from direct patient care.

*Also on the point of less serious condition*, *part of the [regional hospital] rotation was at the [community health centre] and that was lovely as well because you get to see your runny noses*, *and your scratchy throats and things like that. And things that*, *having been in [CAH] for all this time*, *you wouldn’t actually think people come to a facility for that*, *but you do because these are an illness of sort. So*, *gaining an approach to*, *um*, *actually taking time to evaluate someone’s problem … So that was nice getting to work with [nursing] sisters and um learning how to approach those things as if they were as important as things you would see at [CAH]. (FG2)*

One of the starkest differences was rooted in how the students experienced how members of the healthcare teams worked with one another. One student stated:

*I now know … how colleagues treat one another … coming back to [CAH] was kind of depressing. (FG5)*

### A personal learning journey

Students’ reflections suggest that apart from learning more about the primary healthcare environment and honing their clinical skills, they also learned about themselves, felt that they had ‘*grown up*’, and were much more confident about themselves and their role as future doctors. Many described feeling affirmed, feeling that they were adding value, ‘*doing something worthwhile*’ - something that could make a difference and bring about change.

*We definitely felt like we added value*, *when we were there*, *and I think that’s a really nice feeling to have. Sometimes [at CAH] I don’t feel like I was valued. Just also because there’s so many registrars with so many consultants and so many very qualified people making decisions of far above you. (FG1)*

Students also had to deal with the healthcare system head-on. In this context, several students expressed frustration about what they saw at some sites, relating stories of patients who did not get the treatment they deserved, often because the healthcare system had let them down. They described feeling powerless to respond, observing how doctors worked in a busy emergency centre ‘*balancing neglect with speed*’. While they felt ‘*empowered*’ taking on new responsibilities, some emphasized they often felt uncertain as to whether they were making the right decisions, with the issue of prescribing medicines being a specific area in which they wanted more guidance. A particular challenge highlighted by some students was the dilemma they experienced when what they saw at a particular site contradicted what they had been taught. Here some articulated a need for opportunities for debriefing and support.

*Look*, *I saw quite a few bad examples of medicine in [rural hospital]. And I think it just kind of drove home to me like the standard of which we can access training at Stellenbosch is really good. And it kind of taught me that like what I want to not do in terms of*, *like*, *when I graduate. When I become a doctor. … it was rewarding to kind of be directly involved in patient care to affect their management to advocate them [but it] was also very exhausting. … [IDEAL] opened that door*, *in to*, *like*, *systemic*, *the systemic burden of healthcare and kind of like it has kind of taught me to be a bit more proactive in my own self-care*, *I think*, *in terms of like making sure that if I feel this way now*, *I can sustain what I want to do later on … and also not to take on too much*, *just in that sense. So it was definitely a personal journey. (FG2)*

*I think it was very frustrating not getting patients the help they needed … great I know all about these amazing biological therapies or even about these surgeries we could perform*, *the alternative antibiotics*, *then why do I know about all this*, *why do training*, *why do I put all this effort and then you tell me we’re still not seeing the patient [due to COVID-19] or [referral hospital] just refuses to take them … I hated that*, *I hated the feeling of powerlessness and some of the situations as well. It was probably one or the other. On the one hand you feel like you make a difference or on the other you feel like nothing that I can do will make a difference. (FG4)*

Individual students spoke about how their IDEAL experience had convinced them that it would be worthwhile to work in a rural environment or specialise in Family Medicine. One student shared that they felt that what they were doing made sense and was of value. Specifically, students described having learned ‘*life lessons*’, such as that some patients will die and ‘*it’s not your fault*’.

*And by the end of it*, *it just sort of brought home the idea that this [career] is definitely something that you can make a difference with … having the chance to be left up to your [own] devices and be able to formulate a plan and have that plan be to the best of the patient is very gratifying and it sort of stays with you*, *that in the midst of when you might feel a bit despondent in this career and having chosen this path*, *it can sort of be the light at the end of the tunnel and make things remind you exactly as to why you chose [it]. (FG2)*

*And then I mean while you were there having all these contradictory thoughts*, *it’s like*, *yah*, *the specialist life that you had imagined for yourself*, *in Cape Town CBD*, *suddenly feels so futile and now you just wanna go to [the rural area] and make a difference there. Then you get back here and it’s like you wanna go back there. So it has broadened my horizons let me put it like that. (FG4)*

### Person, participation and place

We went on to deductively test the themes presented above against previous work that explored the transformative learning experience of being and becoming a doctor in the clinical learning environment [17]. This analysis suggested that the dimensions of person, participation, and place held true for our respondents, and that the IDEAL rotation represented a rite of passage towards becoming a healthcare professional (Table 1).

**Table 1:** Summary of key findings mapped on to the dimensions of person, participation and place

Dimension	Key findings
Person	Confident, but not always entirely so Changed world view Discerning Revisits former goals A very personal journey
Participation	Being part of the team Valuing ‘the team’ Relationships A humane experience
Place	Opportunities for hands on self-directed learning Authentic clinical environment Where the focus is all about the patient, but with a concern about the system and nature of patient care Enables longitudinal exposure to patients – learn from the patient Characterised by availability and disposition of the supervisors

## Discussion

Our specific research question was what factors influence learning by students undertaking the IDEAL rotation at multiple sites on a distributed training platform? Our intention with this work is to reflect on the lessons that student learning experiences offer for continuing and extended training on a distributed training platform.

In summary, and despite some caveats, the students’ responses attest to a learning experience that was pedagogically enriching on both an inter- and intra-personal level. Being away from the exposure that the central academic hospital offers meant that students could encounter patients and contexts that are equally important for their learning and preparedness as future doctors [21]. These findings offer insights into how distributed rural clinical learning spaces influence the ‘being and becoming’ [17] of senior medical students.

Distributed site training, where students are immersed in the healthcare team and take responsibility for supervised patient management, leads to reported improvements in clinical skills [22]. However, the findings as presented suggest that something much more profound was taking place through the multi-layered learning experience. The extent of transformative learning through the IDEAL rotation, that the students interviewed clearly described, reinforces the domain of **person** as a critical element in the transition to becoming a doctor, in the ‘personal learning journey’ and ‘collegial learning experience’ themes. While this transition was not particularly an intended outcome for IDEAL, it is an intended outcome of the student internship as a whole [17, 23]. What was striking in these results is the comparison that these students drew between previous clinical rotations and this one in enabling a sense of that transition. It has been suggested that an important contributor to this for students may be a greater responsibility both in clinical practice and in directing their own learning [24]. Most striking in our findings is the many references to how IDEAL shifted students’ thinking and perspectives about healthcare and their role in it, which are manifestations of the emergence of a professional identity that is core to transformation [25, 26].

Key to the engagement that leads to transformation is **participation** in a community of clinical practice. Students describe the teams they were part of as being very experienced and humanizing. Even more, they describe how, while being part of these clinical practice communities, they became acutely mindful of the central role of the patient – not the patient as a disease but the patient as a person, a whole person within a family and community, and the delight of being able to experience meaningful connections with patients and a breadth of healthcare professionals. This is best described as relationship-based learning, which was traditionally how critical knowledge, skills, and attitudes were transferred to new generations of doctors [27]. Developing relationships is still a key factor in the learning process during the clinical training of medical students [28]. Relationships have been shown to underpin effective clinical supervision [29], influence the amount of teaching that takes place [30], affect the usefulness of feedback [31], and contribute to professional identity formation [32], all of which are illustrated by the students’ descriptions of their experiences during IDEAL.

Ultimately, our findings demonstrate the important of **place** in terms of the context in which training occurs. The learning experiences that the students describe arise because of a change of context. The advantage of distributed training is that it exposes students to contexts, in which they experience different informal learning and socialisation processes [33]. Although it may be difficult to define, “context matters in medical education” [33] and we need to take it seriously when thinking about where and how we place our students. With support from our findings, it can be argued that context is as important as content in developing professional identity. How we structure curricula matters – and if we hope to design for transformative learning, we need to move from a notion of context that values learning from, and in, specific places or communities to an approach that includes a context-critical analysis of power, histories and experiences, which is essential for a critical pedagogy of place [34].

While standardisation of clinical experience may be something of a Holy Grail in medical education, there is good evidence and a strong argument for the value of contextual diversity in training [35]. It has been argued that there is aways a contextual curriculum in clinical training that influences intentional and unintentional learning, similar to the notion of a hidden curriculum [36]. It requires us, as curriculum planners, to be focused on the strengths and possibilities of the context in which we place our students when attempting to support transformative learning. We must acknowledge that students in IDEAL also reported difficult experiences. Thus, when thinking of IDEAL becoming a permanent clinical rotation in the curriculum, we need to be aware of the responsibility we have to prepare and support students in these distributed training contexts to avoid harm through transformation [37]. It is vital that as we prepare students for the real world of healthcare that they will enter as graduates, we need to assist them in this transition.

Perhaps the most important aspect of the context that arises from this work, which the students can teach us, is that in many students’ experience, the contexts that IDEAL placed them in were often those in which patients were perceived to be at the center of care. Most students come into the medical profession not only because they have achieved well academically but also because they have a desire to serve humankind in one way or another. Unfortunately, the latter is often diminished with the focus on the science of medicine and the perhaps narrowly defined clinical knowledge that students need to master [38] to achieve at the high level they have been used to. The art of medicine, in which patients are at the center, and relationships are the key to practice, is easily lost. In addition, there are growing calls globally for medical graduates who are more socially responsive to the healthcare needs of the societies they will eventually serve [39]. Ensuring training occurs in contexts in which students are reminded of their own altruistic intent and their own humanity should be preeminent in our planning if we wish to see our graduates embody the change we hope to see in the health service. If we reflect back to the model of person, participation and place, it does appear that each of these dimensions should be taken into account when planning for similar clinical learning innovations. Equally, it could be of value to explicitly share these with students – that the placement will offer certain affordances that will facilitate their authentic participation while also enabling their own personal growth.

### Limitations

Only a small group of students volunteered to participate in the study. The extent to which they reflected the experiences of their class is unknown; however, the students all came from different sites and a variety of backgrounds. Experiences shared reflected what we received in formal and informal feedback from students, learning facilitators and wellness supporters. The interviews were conducted by a senior academic (IC) involved in planning and implementing the rotation; he only had supervisory contact with a small group of students in IDEAL as a learning facilitator, none of whom participated in the interviews, and he is not involved in any of the final year rotations in the CAH. The research team represented a range of diverse people involved in IDEAL in different ways and with a range of roles, including one who was a student in the first cohort (LS); this provided us with a unique insider perspective that we could bring to the research.

## Conclusions

Reflecting on the lessons learned through implementation of IDEAL gave impetus to curricular renewal in the FMHS. Firstly, the IDEAL rotation was included as an ongoing part of the curriculum post-COVID-19 [9]; in other words, the revised structure of the MBChB programme adopted at that time has continued, because of its added value. Secondly, this will be incorporated into a new final year curriculum to be implemented in 2027, as part of a whole programme renewal process, when it is planned that students will spend 36 weeks on the distributed training platform [40]. We have also learnt from student experiences about the need to continue striving to provide psychological and learning safety. Thus, an important part of ongoing planning is to optimize support for students, in the context of student feedback and despite resource constraints; more support by local clinicians is now in place, but this is a vital element in ongoing research on the IDEAL rotation.

The success of the intervention was not a matter of chance but rather based on many years of experience in developing and testing the kind of model that was used [10, 24, 41] and of researching evidence for the model [15]. In addition, critical relationships had been built with health service partners over many years, enabling us to pivot very quickly [9, 42]. In developing a model for distributed training in health professions education we have come to realize that relationships need to be at the center of everything that we do [11], just as they should be at the center of our learning and teaching.

## Electronic supplementary material

Below is the link to the electronic supplementary material.

Supplementary Material 1

## Data Availability

No datasets were generated or analysed during the current study.

## Footnotes

1 The FMHS in partnership with public health facilities uses up to 100 different sites for clinical training. These are collectively called the clinical training platform.
