# Supplementary material for: It’s all about the patients: a shift in medical students’ approach to learning during a novel distributed integrated clinical rotation
Source: BMC Med Educ. 2024 Oct 15;24:1145. doi: 10.1186/s12909-024-06112-8 (PMC11475787; doi:10.1186/s12909-024-06112-8)
Supplement: Supplementary file 1 — Supplementary Material 1 [file 12909_2024_6112_MOESM1_ESM.pdf]

IDEAL Rotation Research

Interview guide for Focus groups

Introduction:

- Introduction of the Interviewer and students to each other.
- Setting the scene: explaining the process.
- Ensuring everyone has consented, is comfortable and is ready to proceed.
- Answering any questions in the group.

Questions

The following questions will be used as a guide for directing the conversation in the group, with probes, reflection and summarizing being used to ensure that issues are explored fully.

1. Looking back, what stands out for you from your experience of IDEAL?
    a. What was unique or memorable about that experience?
    b. What was most enjoyable?
    c. What was most helpful?
    d. What was disappointing about it?
2. How did you find the experience of learning in IDEAL?
    a. Was there any change in the way that you learned? If yes, what led to the change? Do you think it will enhance your approach to learning going forward?
    b. Do you feel that you learned more or less than in other rotations? Can you give examples?
    c. Are there particular things that you learned about learning? Can you give examples?
3. What has been the impact of IDEAL on you, if any?
    a. Has this made any difference to the way you think about yourself or your patients, or your career?
    b. Are you doing anything differently in your current rotations because of IDEAL?
    c. Has there been an impact on your clinical skills?
4. Is there anything else anyone wants to add regarding IDEAL?
